# Fluid therapy for septic shock resuscitation: which fluid should be used?

**DOI:** 10.1590/S1679-45082015RW3273

**Published:** 2015

**Authors:** Thiago Domingos Corrêa, Leonardo Lima Rocha, Camila Menezes Souza Pessoa, Eliézer Silva, Murillo Santucci Cesar de Assuncao

**Affiliations:** 1 Hospital Israelita Albert Einstein, São Paulo, SP, Brazil.

**Keywords:** Schock, septic, Ressuscitation/methods, Fluid therapy, Colloids, Hydroxyethil starch derivatives, Albumins

## Abstract

Early resuscitation of septic shock patients reduces the sepsis-related morbidity and mortality. The main goals of septic shock resuscitation include volemic expansion, maintenance of adequate tissue perfusion and oxygen delivery, guided by central venous pressure, mean arterial pressure, mixed or central venous oxygen saturation and arterial lactate levels. An aggressive fluid resuscitation, possibly in association with vasopressors, inotropes and red blood cell concentrate transfusion may be necessary to achieve those hemodynamic goals. Nonetheless, even though fluid administration is one of the most common interventions offered to critically ill patients, the most appropriate type of fluid to be used remains controversial. According to recently published clinical trials, crystalloid solutions seem to be the most appropriate type of fluids for initial resuscitation of septic shock patients. Balanced crystalloids have theoretical advantages over the classic solutions, but there is not enough evidence to indicate it as first-line treatment. Additionally, when large amounts of fluids are necessary to restore the hemodynamic stability, albumin solutions may be a safe and effective alternative. Hydroxyethyl starches solutions must be avoided in septic patients due to the increased risk of acute renal failure, increased need for renal replacement therapy and increased mortality. Our objective was to present a narrative review of the literature regarding the major types of fluids and their main drawbacks in the initial resuscitation of the septic shock patients.

## INTRODUCTION

Septic shock is defined as a systemic inflammatory response syndrome, triggered by an infection associated with refractory hypotension, despite a fluid load of 30mL/kg of body weight.^(1)^ Septic shock remains a major cause of morbidity and mortality among critically ill patients, with a mortality rate between 20 to 45%.^(2-5)^ Even though most deaths in septic shock have been attributed to progression to multiple organ failure syndrome, the puzzle concerning sepsis-related organ dysfunction and failure remains unsolved.^(6)^ Systemic inflammation, microvascular abnormalities, tissue hypoperfusion, mitochondrial dysfunction and the therapeutic interventions made on septic patients may contribute to progression towards organ failure and death.^(6)^

The landmark of septic shock is systemic vasodilation, with different levels of hypovolemia.^(7)^ Fluid administration is the first-line intervention to restore the systemic hemodynamics and increase oxygen delivery to match oxygen demand in septic patients.^(7) ^According to the Surviving Sepsis Campaign Guidelines, septic patients presenting tissue hypoperfusion, hypotension or signs of hypovolemia and admitted to the emergency department must receive an initial fluid load with 30mL/kg of body weight of crystalloids.^(1)^ Patients with sustained hypotension (*i.e., *mean arterial blood pressure – MAP <65mmHg) or those with initial arterial lactate concentration >4.0mmol/L, must be resuscitated following the goal-directed therapy protocol, *i.e*., resuscitation guided by central venous pressure (CVP), MAP, and central venous oxygen saturation (ScvO_2_) or mixed venous oxygen saturation (SvO_2_).^(1) ^The goals to be achieved during the initial 6 hour resuscitation include: a CVP between 8 and 12mmHg, in spontaneous breathing, or between 12 and 15mmHg, in patients under mechanical ventilation; a MAP >65mmHg; and ScvO_2_ or SvO_2_ >70% and 65%, respectively. Alternatively, a lactate clearance >10% may be target during this first 6 hours, in place of the ScvO_2_, in patients with no central venous catheter.^(8)^ Fluids, vasopressors, inotropes and red blood cells transfusion are the therapeutic interventions available at the bedside for critical care and emergency care physicians to achieve those hemodynamic goals. It is important to emphasize that many patients can be fully resuscitated only by early receiving the correct type and amount of intravenous fluids.

The question concerning which fluids should be used during the initial hours of septic patients has been a matter of debate for decades and up to now, there has been no consensus over which type of fluid is the most appropriate to be used in this context.^(9)^ Therefore, our objective was to present a narrative review of the literature regarding the major types of fluids and their main drawbacks for in the initial resuscitation of the septic shock patients.

## WHY FLUIDS ARE GIVEN?

The reason to offer fluids to septic shock patients may be justified based on vascular bed changes. Inflammatory mediators act on endothelial cells promoting vasodilation, which results in a relative hypovolemia, *i.e*., loss in continent and content ratio.^(10)^ Therefore, an adequate fluid replacement is crucial to maintain the perfusion pressure and, most importantly, blood flow to the tissues.^(11) ^Tissue perfusion, systemic blood flow and oxygenation can be achieved by restoring the intravascular compartment through fluid administration.^(12)^

Fluids should be administered based on two assumptions: in the presence of impaired tissue perfusion (stagnant hypoxia) requiring blood flow augmentation and in the presence of fluid responsiveness, that is, when fluid administration will boost cardiac output.^(13)^ Both hypovolemia and fluid overload can be deleterious for critically ill patients. Therefore, whenever feasible it is advisable to address fluid responsiveness before prescribing fluids as well as to avoid fluids infusion in the patients to whom those assumptions do not apply. Inotropes should be considered to improve tissue oxygenation in patients requiring an increased blood flow but who are no longer responsive to fluid administration.^(1)^

Early recognition and prompt treatment are crucial to improve survival in septic shock patients.^(14)^ Early onset of resuscitation can restore the oxygen delivery, reverse tissue hypoxia and minimize the progression to cell and mitochondrial dysfunction and the establishment of multiple organ failure syndrome secondary to systemic inflammation and tissue hypoperfusion.^(15) ^Alongside with fluid therapy, infection control with early adequate antibiotics administration is essential to mitigate damage secondary to the inflammatory response.^(16)^ Nevertheless, tissue dysoxia can develop even after adequate fluid resuscitation, depending on the intensity of systemic inflammation and the severity of disease.

## TYPES OF FLUIDS

Resuscitation fluids can be divided into two broad categories: crystalloids and colloids (Tables 1 and 2).^(9)^ Different types of solutions can have specific capacity of volume expansion, duration of effect, impact on vascular integrity, acid-base balance, inflammatory response, changes in red blood cell rheology and hemostasis.^(9)^ These alterations can result in beneficial or harmful effects, depending on the characteristics of patients and fluids. In the following sections, we will discuss the main available types of fluids for septic shock resuscitation.

**Table 1:** The main crystalloid solutions and their composition(9)

Solutions/characteristics	Osmolality (mOsm/L)	pH	Sodium (mEq/L)	Chloride (mEq/L)	Potassium (mEq/L)	Calcium (mEq/L)	Magnesium (mEq/L)	Buffers (mEq/L)
Plasma	290	7.4	140	103	4	4	2	Bicarbonate (24)
Normal saline (0.9% NaCl)	308	5.7	154	154	0	0	0	0
Ringer’s injection	309	5.8	147	156	4	4	0	0
Ringer lactate	273	6.5	130	109	4	3	0	Lactate (28)
Ringer acetate	275	6.7	131	109	4	3	0	Acetate (28)
Plasma-Lyte	295	7.4	140	98	5	0	3	Acetate (28)Gluconate (23)

**Table 2:** The main colloidal solutions and their composition(9)

Solutions/characteristics	Albumin		Hydroxyethyl starch		Dextran		Gelatins
Solution concentration	4%, 5%	20%, 25%	6%, 10% pentastarch	6% hetastarch	10% Dextran 40	3% Dextran 60 6% Dextran 70	
Molecular weight	69		100-450		40-70		30-35
Osmolality (mOsm/L)	300	1.500	300-326		280-324		300-350
Oncotic pressure (mmHg)	19-30	74-120	23-82		20-60		25-42
Plasmatic expansion (%)	70-100	200-300	100-160		100-200	80-140	70-100
Duration of plasmatic expansion (h)	≤24		≤12	≤4-36	≤4-6	≤8-24	≤4-6
Plasma half-life (h)	16-24		2-12		2	~24	~2-9
Possible adverse effects	High cost, risk of infection and anaphylactic reactions		Impairment coagulation, pruritus, acute kidney failure, and anaphylactic reactions		Changes in blood viscosity, coagulopathy, renal dysfunction, and anaphylactic reactions		Hypercalcemia and anaphylactic reactions

## CRYSTALLOIDS

Crystalloid solutions have been recommended as a first choice to resuscitate septic shock patients and nowadays they are the most used type of fluids in the Unites States.^(1,9)^ “Crystalloid” is the most popular term used to refer to solutions containing water, inorganic ions and small organic molecules. Crystalloids are composed of glucose or sodium chloride solutions, and can be hypotonic, isotonic or hypertonic. Some of them can have other components, such as potassium or calcium, and can use some buffers as lactate or acetate to become plasma-like.^(9)^

Normal saline (0.9% NaCl) is considered an isotonic solution, with osmolality closer to the plasma osmolality (287mOsm/kg) and it contains a sodium concentration of 154mEq/L and a chloride concentration of 154mEq/L, which is 1.5-fold higher than the physiologic serum concentration of chloride. This is the reason for normal saline being considered a non-balanced solution.^(9)^ Because normal saline has a higher chloride concentration, large volume infusions can promote hyperchloremic acidosis, also known as dilution hyperchloremic acidosis, which can be explained by the strong ion difference (SID) approach.^(17)^

SID is defined as the difference between cations and anions dissociated in plasma.^(17)^ In a 70-kg healthy man, the sodium and chloride plasma concentrations are approximately 140mEq/L and 100mEq/L, respectively, and the total body water is around 42L. Therefore, in healthy individuals, the cation concentration exceeds that of anions, resulting in a difference of 40mEq/L (plama SID=40mEq/L). Under those conditions, the total body sodium and chloride concentrations are, respectively, 5,880mEq and 4,200mEq. If that individual receives 10L of normal saline, it will add 1,540mEq in plasma sodium and 1,000mEq in plasma chloride, resulting in final total body sodium and chloride of 7,420mEq and 5,200mEq, respectively. The total body water will be also increased from 42 to 52L. Accordingly, the SID is reduced to 32mEq/L, as sodium and chloride concentrations increases to 142.7 and to 110mEq/L, respectively.^(18,19)^ This occurs because normal saline contains strong cations and strong anions in the same quantity, *i.e*., its SID is zero. Thus, infusion of normal saline will reduce the SID of plasma and, therefore, decrease the plasma pH. In general, acidosis is mild to moderate, base excess variation is not higher than -10mEq/L and rarely the pH reaches less than 7.30 after respiratory compensation.^(20)^ If normal saline infusion is interrupted, the effects are expected to be transitory and reversible within 48 hours.^(21)^

Besides the hyperchloremic acidosis, large amounts of normal saline infusion can compromise coagulation, kidney function and the immunologic response.^(22)^ Dilutional coagulopathy occurs because all coagulation factors will be diluted by infused bulk normal saline, increasing risk of bleeding.^(22)^ Moreover, there is a growing body of evidence showing that normal normal saline can impair renal function. In experimental animal studies, hyperchloremia induced by normal saline showed to diminish renal blood flow and promote renal vasoconstriction.^(23,24)^

Balanced solutions have been proposed as an alternative to normal saline.^(25)^ A solution can be considered ideally balanced when it is normotonic with a SID of 24mEq/L.^(26)^ This can be achieved by removing 24mEq/L of chloride from 0.9% sodium chloride solution and replacing it with bicarbonate or organic anions, which quickly disappear after infusion, such as lactate or acetate.^(26)^ Considering the previously described adverse events related to unbalanced solution, balanced solutions might be the ideal solution for the resuscitation of critically ill patients.

The most common used balance solutions include Ringer’s injection, Ringer Lactate, Ringer Acetate and Plasma-Lyte. The Ringer Lactate was developed in the beginning of 1930s, by adding sodium lactate to Ringer solution as a buffer, reducing its chloride concentration (109mEq/L) when compared to Ringer’s injection solution (Table 1). Ringer Lactate is a mild hypotonic solution (273mOsm/kg) and has potassium and calcium in its composition. Concerns that large amounts of Ringer Lactate infusion could increase plasma lactate levels in critically ill patients led the lactate buffer to be replaced by acetate in order to create Ringer Acetate. The composition of Ringer Lactate and acetate is almost identical with the exception of the added buffer (lactate or acetate). Plasma-Lyte is another balanced solution with osmolality of 295mOsm/L, sodium concentration of 140mEq/L and chloride concentration of 98mEq/L. Other electrolytes and buffers making up this solution are potassium, magnesium, acetate and gluconate. In patients with impaired kidney function, this kind of solution should be avoided due to the risk of hyperkalemia (Table 1).

It was demonstrated that a chloride-restrictive strategy in critically ill patients was associated with a significant decrease in the incidence of acute kidney injury and use of renal replacement therapy.^(27)^ Additionally, a large retrospective cohort study involving 53,448 septic patients showed that resuscitation with balanced fluids, in comparison to non-balanced solutions, decreases the risk of in-hospital death (relative risk – RR=0.86; 95% confidence interval – 95%CI: 0.78-0.94; p=0.001). However, no significant differences in the incidence of acute renal failure, the need of renal replacement therapy, and hospital and intensive care unit (ICU) lengths of stay were reported.^(25)^

Nevertheless the level of evidence to support the use of balanced solutions in clinical practice is weak.^(28)^ A recent meta-analysis suggested that resuscitation with balanced crystalloids in comparison to unbalanced solution (normal saline 0.9%) may be associated with lower mortality rate (*odds ratio* – OR=0.78; 95%CI: 0.58-1.05).^(28)^ In this context, a large randomized trial comparing balanced and unbalanced solutions for septic shock resuscitation is still needed.^(28)^

## COLLOIDS

Colloids are defined as homogenous non-crystalloid substances consisting of large molecules or ultramicroscopic particles of one substance dispersed through a second substance molecule with a high molecular weight.^(29)^ Those fluids have a relatively higher duration and capacity of intravascular expansion with lower volumes, *i.e*., a higher oncotic pressure when compared to crystalloids. Colloids are not able to cross the semi impermeable vascular membrane due to their high molecular weight.^(29)^

There are two kinds of colloids: natural and semisynthetic colloids. Human albumin in normal saline is the reference colloidal solution and it represents a natural colloid derived from human plasma. Semisynthetic colloids, by contrast, consist of derivatives of three main groups of molecules: gelatins, dextrans and starches (Table 2). To produce a colloid, these molecules are suspended in a solvent, which can be an isotonic or hypertonic normal saline, hypertonic glucose or an isotonic balanced electrolyte solution. Isotonic normal saline is the most common solvent used in colloidal solutions. Due to the scope of this review, we will focus on starch solutions and albumin.

### Hydroxyethyl starch

Hydroxyethyl starch (HES), a synthetic solution made by manipulating waxy or potato amylopectin (a multi-branched glucose polymer), has become some of the most frequently used colloidal plasma expanders worldwide, mainly due to their lower cost when compared to albumin.^(30)^ Nowadays, HES are being avoided in the treatment of critically ill patients, specifically in those with sepsis. Recent clinical data indicate that colloids do not improve patient outcomes and may be harmful depending on the setting and type of colloid.^(31,32)^

HES are identified by three numbers, *e.g*. 10% HES 200/0.5 or 6% HES 130/0.4. They are classified according to the mean molecular weight (range: 70 to 670 kiloDalton) and the frequency of hydroxyethyl groups per glucose monomer (range: 0.4 to 0.7).^(33)^ The first number indicates the solution concentration, the second represents the mean molecular weight expressed in kiloDalton (kDa), and the third and most significant one is the molar substitution (MS).^(33)^

HES have a varying number of hydroxyethyl residues attached to the anhydrous glucose particles within the polymer. This substitution increases the solubility of starch in water and, to a varying degree, inhibits the rate of degradation of the starch polymer by amylases.^(33)^ Those parameters are highly relevant to the pharmacokinetics of HES. The half-life of a starch solution depends on its molecular weight, degree of substitution, and the proportion of hydroxyethyl groups in the C2 carbon when compared with the C6 carbon of the glucose monomer.^(34)^ Hydroxyethyl groups at the position of the C2 atom inhibit the access of alpha-amylase to the substrate more effectively than hydroxyethyl groups at the C6 position. Hence, HES produced with high C2/C6 ratios are expected to be more slowly degraded.^(34)^ In general, HES is used for restrictive fluid strategy due to a high plasma expansion capacity with lower volume administration.

A prospective multicenter clinical trial was performed to address the frequency of acute renal failure in severe sepsis and septic shock patients resuscitated with 6% HES (200kDa, 0.60; 0.66 substitution) or 3% fluid-modified gelatin.^(35) ^The frequencies of acute renal failure, oliguria and the peak serum creatinine concentration were significantly higher in the HES group in comparison to the gelatin group.^(35)^ In this study, HES resuscitation was found to be an independent risk factor for acute renal failure (OR=2.57; 95%CI: 1.13-5.83; p=0.026) in severe sepsis or septic shock patients.^(35) ^Other studies evaluated starches with a high or intermediate molecular weight (200 or 450kDa) and a higher degree of molar substitution (0.5 to 0.7) and showed a higher incidence of renal failure or bleeding complications.^(36)^

It was advocated that the third generation HES, with a lower molecular weight and lower degree of molar substitution, would have a safer profile and, therefore, would be associated to a lower incidence of adverse events (mainly bleeding complications and acute kidney injury). Hence, they could be used to treat critically ill patients. Nonetheless, this hypothesis was not confirmed in the most recent clinical trials.^(37-39)^ In those studies, resuscitation with a third generation of HES was associated with an increased risk of death, acute renal failure and the need of renal replacement therapy, especially among the septic patients.^(37-39)^ The reported results were quite similar regardless of whether potato- or maize-derived starch were compared.^(40)^ In sum, the most recent literature does not support the use of HES during the resuscitation of severe sepsis and septic shock patients.

### Albumin

Albumin solutions are used worldwide to treat critically ill patients. A meta-analysis carried out in 1998 associated albumin usage to high mortality rate.^(41)^ In this context, safety of albumin use was questioned until the SAFE study (Saline versus Albumin Fluid Evaluation) was published, which showed that albumin solutions, in comparison to crystalloids, did not increase mortality.^(42)^

Albumin administration can be justified based on its physiological effects, primarily binding and transportation of various substances (such as drugs and hormones) in the blood; antioxidant properties, nitric oxide modulation; and buffer capacity, which may be of particular relevance in critically ill patients, and not only to regulate osmotic pressure.^(43)^ Also, low serum albumin levels, a common finding in critically ill patients, were associated to worse outcomes.^(44)^ On the other hand, there are some limitations for a broader use of albumin solutions: their high cost, potential risk of microorganisms transmission and allergic effects when compared to crystalloids.^(44)^ So far, no randomized clinical trial has showed a real benefit of albumin administration. Actually, it could be noticed that some subpopulations, such as those with traumatic brain injury, can have an increased risk of death when receiving albumin solutions.^(45)^

A subgroup analysis among septic shock patients enrolled in the SAFE study showed a trend on mortality reduction in favor to albumin in comparison to normal saline (adjusted OR=0.71; 95%CI: 0.52-0.97; p=0.03).^(46)^ More recently, the ALBIOS study (Volume Replacement With Albumin in Severe Sepsis) randomized 1,818 severe sepsis and septic shock patients to receive either 300mL of 20% albumin plus crystalloid or to receive crystalloid alone from randomization until day 28, or ICU discharge, aiming to maintain serum albumin ≥30g/L.^(47) ^Although more patients reached the target MAP within 6 hours in the albumin group when compared to crystalloids group, neither the 28-day mortality nor the 90-day mortality rate (RR=0.94; 95%CI: 0.85-1.05; p=0.29) differed between the groups.^(47)^

It can be concluded that a clinical trial comparing a 4% albumin solution with crystalloids to resuscitate septic shock patients is required. For instance, albumin solution is the only colloidal solution endorsed by the Surviving Sepsis Campaign guidelines for septic patients who are not responding to crystalloid infusion.^(1)^

## CONCLUSION

There is no consensus over which type of fluid should be used as first-line therapy to resuscitate septic shock patients. The body of evidence shows that crystalloids solutions, whether balanced or not, are the most advisable choice. Hypo-oncotic albumin solutions can be used as an alternative for those who need large amounts of fluids during the initial resuscitation phase. The hydroxyethyl starches should not be prescribed to this population due to possible deleterious effects. Further evidence on the use of albumin and balanced solutions is required.
