# Supplementary material for: Fluid therapy for septic shock resuscitation: which fluid should be used?
Source: Einstein (Sao Paulo). 2015 Jul-Sep;13(3):462–8. doi: 10.1590/S1679-45082015RW3273 (PMC4943797; doi:10.1590/S1679-45082015RW3273)
Supplement: Translation

# Translation: Fluidoterapia para a ressuscitação no choque séptico: qual tipo de fluido deve ser utilizado?

**Authors:** Thiago Domingos Corrêa, Leonardo Lima Rocha, Camila Menezes Souza Pessoa, Eliézer Silva, Murillo Santucci Cesar de Assuncao

**Affiliations:** 1 Hospital Israelita Albert Einstein − São Paulo, SP, Brasil.

**Keywords:** Choque séptico, Ressuscitação/métodos, Hidratação, Coloides, Derivados de hidroxietil amido, Albuminas

## Abstract

A ressuscitação precoce de pacientes com choque séptico tem o potencial de reduzir sua morbidade e mortalidade. Os objetivos principais da ressuscitação no choque séptico incluem expansão volêmica, manutenção da perfusão tecidual e da oferta de oxigênio para os tecidos, guiados pela pressão venosa central, pressão arterial média, saturação venosa mista ou central de oxigênio e lactato arterial. Uma ressuscitação agressiva com fluidos, possivelmente em associação com vasopressores, inotrópicos e transfusão de concentrado de hemácias, pode ser necessária para atingir estes objetivos hemodinâmicos. Todavia, embora a administração de fluidos seja uma das intervenções mais comumente realizada em pacientes graves, o tipo de fluido mais apropriado para ser utilizado permanece controverso e incerto. De acordo com os estudos clínicos mais recentes, os cristaloides são os fluidos de escolha para serem utilizados na ressuscitação inicial de pacientes com choque séptico. As soluções cristaloides balanceadas possuem vantagens teóricas em relação as não balanceadas, porém ainda não há evidências suficientes para indicá-las como tratamento de primeira escolha. Além disso, albumina humana parece ser uma alternativa segura e efetiva quando grandes quantidades de fluidos são necessárias para o restabelecimento da estabilidade hemodinâmica. O uso de soluções de hidroxetilamido deve ser evitado em pacientes sépticos, devido ao maior risco de desenvolvimento de insuficiência renal aguda, necessidade de terapia de substituição renal e aumento de mortalidade. O objetivo deste estudo foi apresentar uma revisão narrativa da literatura sobre os principais tipos de fluidos e os problemas mais importantes na ressuscitação inicial de pacientes com choque séptico.

## INTRODUÇÃO

O choque séptico é definido como síndrome de resposta inflamatória sistêmica, desencadeada por uma infecção associada à hipotensão refratária, mesmo com a administração de 30mL/kg de peso corporal de fluidos.^(1)^ Trata-se de uma importante causa de morbidade e mortalidade entre pacientes graves, com taxa de mortalidade variando entre 20 e 45%.^(2-5)^ Apesar de a maioria das mortes por choque séptico serem atribuídas à evolução para síndrome da falência múltipla de órgãos, a fisiopatologia da disfunção e da falência de órgãos, relacionada à sepse, ainda não foi elucidada completamente.^(6)^ Inflamação sistêmica, alterações microvasculares, hipoperfusão tecidual, disfunção mitocondrial e intervenções terapêuticas realizadas no paciente séptico podem contribuir para a progressão para falência de órgãos e morte.^(6)^

A característica principal do choque séptico é a vasodilatação sistêmica com diferentes graus de hipovolemia.^(7)^ A administração de fluidos é a intervenção de primeira escolha para restaurar a hemodinâmica e aumentar a oferta de oxigênio, de modo a atender à demanda por oxigênio em pacientes sépticos.^(7)^ De acordo com as Diretrizes da Campanha Sobrevivendo à Sepse, pacientes sépticos que apresentem hipoperfusão tecidual, hipotensão ou sinais de hipovolemia admitidos no pronto-atendimento devem receber um volume inicial de fluidos cristaloides de 30mL/kg de peso corporal.^(1)^ Pacientes com hipotensão sustentada (ou seja, pressão arterial média − PAM <65mmHg) ou com concentração inicial de lactato arterial >4,0mmol/L devem ser ressuscitados seguindo o protocolo de terapia guiada por metas: ressuscitação orientada pela pressão venosa central (PVC), PAM e saturação venosa central (ScvO_2_) ou venosa mista de oxigênio (SvO_2_).^(1) ^As metas a serem atingidas durante a ressuscitação inicial de 6 horas incluem PVC entre 8 e 12mmHg, em respiração espontânea, ou entre 12 e 15mmHg, em pacientes em ventilação mecânica; PAM >65mmHg; e ScvO_2_ ou SvO_2_ >70% e 65%, respectivamente. Como alternativa, em vez do ScvO_2_, pode-se utilizar o *clearance* de lactato >10% como meta durante as primeiras 6 horas, para pacientes sem cateter venoso central.^(8)^ Fluidos, vasopressores, agentes inotrópicos e transfusão de hemácias são as intervenções terapêuticas disponíveis à beira do leito para os médicos intensivistas e emergencistas conseguirem as metas hemodinâmicas. É importante enfatizar que muitos pacientes podem ser plenamente ressuscitados apenas recebendo precocemente o tipo e a quantidade corretos de fluido intravenoso.

A questão sobre quais fluidos devem ser utilizados durante as primeiras horas de ressuscitação dos pacientes sépticos é assunto discutido há décadas. Até o momento, não há consenso sobre qual tipo de fluido é o mais adequado para uso neste contexto.^(9)^ Assim, nosso objetivo foi apresentar uma revisão narrativa da literatura relacionada aos principais tipos de fluidos para ressuscitação inicial de pacientes com choque séptico.

## POR QUE ADMINISTRAR FLUIDOS?

Pode-se justificar a oferta de fluidos para pacientes em choque séptico com base nas alterações do leito vascular. Mediadores inflamatórios agem sobre as células endoteliais e promovem vasodilatação, que resulta em hipovolemia relativa, ou seja, em perda na relação continente/conteúdo.^(10)^ Assim, a reposição adequada de fluidos é crucial para a manutenção da pressão de perfusão e, mais importante, do fluxo sanguíneo tecidual.^(11) ^Podem ser conseguidos perfusão tecidual, fluxo sanguíneo sistêmico e oxigenação restaurando-se o compartimento intravascular pela administração de fluidos.^(12)^

Os fluidos devem ser administrados com base em duas premissas: na presença de perfusão tecidual insuficiente (hipóxia estagnante), que exija aumento do fluxo sanguíneo, e na presença de responsividade a fluidos, ou seja, a administração de fluidos aumentará o débito cardíaco.^(13)^ Tanto a hipovolemia quando a sobrecarga de fluidos podem ser deletérias para pacientes graves. Desse modo, aconselha-se avaliar, sempre que possível, a responsividade a fluidos antes de sua prescrição, assim como evitar a infusão de fluidos nos pacientes nos casos em que as premissas não se aplicam. Devem-se considerar os agentes inotrópicos para melhorar a oxigenação tecidual em pacientes que necessitam de maior fluxo sanguíneo, mas que não respondem à administração de fluidos.^(1)^

A identificação precoce e o tratamento imediato são fundamentais para melhorar a sobrevida de pacientes em choque séptico.^(14)^ O início precoce da ressuscitação pode restaurar a oferta de oxigênio, reverter a hipóxia tecidual, e minimizar a progressão para disfunção celular e mitocondrial e para o estabelecimento da síndrome da falência múltipla de órgãos, secundária à inflamação sistêmica e à hipoperfusão tecidual.^(15)^ Além da fluidoterapia, o controle de infecção com administração precoce de antibióticos adequados é essencial para mitigar as lesões secundárias à resposta inflamatória.^(16) ^ No entanto, a disóxia tecidual pode ocorrer mesmo após ressuscitação adequada com fluidos, dependendo da intensidade da inflamação sistêmica e da gravidade da doença.

## TIPOS DE FLUIDOS

Os fluidos para ressuscitação, podem ser divididos em duas grandes categorias: cristaloides e coloides (Tabelas 1 e 2).^(9)^ Diferentes tipos de soluções podem apresentar especificidades em relação à capacidade de expansão volêmica, duração de efeito, impacto sobre integridade vascular, equilíbrio acidobásico, resposta inflamatória, alterações na reologia de hemácias e na hemostase.^(9)^ Essas alterações podem resultar em efeitos benéficos ou deletérios, dependendo das características do paciente e do fluido. Nas seções a seguir, discutiremos os principais tipos de fluidos disponíveis para a ressuscitação em casos de choque séptico.

**Tabela 1:** Principais soluções cristaloides e sua composição(9)

Soluções/características	Osmolalidade (mOsm/L)	pH	Sódio (mEq/L)	Cloreto (mEq/L)	Potássio (mEq/L)	Cálcio (mEq/L)	Magnésio (mEq/L)	Tampões (mEq/L)
Plasma	290	7,4	140	103	4	4	2	Bicarbonato (24)
Solução salina (NaCl 0,9%)	308	5,7	154	154	0	0	0	0
Ringer simples	309	5,8	147	156	4	4	0	0
Ringer lactato	273	6,5	130	109	4	3	0	Lactato (28)
Ringer acetato	275	6,7	131	109	4	3	0	Acetato (28)
Plasma-Lyte	295	7,4	140	98	5	0	3	Acetato (28) Gluconato (23)

**Tabela 2:** Principais soluções coloidais e sua composição(9)

Soluções/ características	Albumina		Hidroxietilamido		Dextran		Gelatinas
Concentração da solução	4%, 5%	20%, 25%	6%, 10% pentastarch	6% hetastarch	10% de Dextran 40	3% de Dextran 60 6% de Dextran 70	
Peso molecular	69		100-450		40-70		30-35
Osmolalidade (mOsm/L)	300	1.500	300-326		280-324		300-350
Pressão oncótica (mmHg)	19-30	74-120	23-82		20-60		25-42
Expansão plasmática (%)	70-100	200-300	100-160		100-200	80-140	70-100
Duração da expansão plasmática (h)	≤24		≤12	≤4-36	≤4-6	≤8-24	≤4-6
Meia-vida plasmática (h)	16-24		2-12		2	~24	~2-9
Possíveis efeitos adversos	Alto custo, risco de infecção e reações anafiláticas		Défice de coagulação, prurido, insuficiência renal aguda e reações anafiláticas		Alterações na viscosidade sanguínea, coagulopatia, disfunção renal e reações anafiláticas		Hipercalcemia e reações anafiláticas

## CRISTALOIDES

Soluções cristaloides são recomendadas como primeira escolha para ressuscitação de pacientes em choque séptico e, atualmente, são o tipo de fluido mais utilizado nos Estados Unidos.^(1,9)^ “Cristaloide” é o termo mais utilizado para se referir a soluções contendo água, íons inorgânicos e pequenas moléculas orgânicas. Essas soluções contêm glicose ou cloreto de sódio e podem ser hipotônicas, isotônicas ou hipertônicas. Algumas podem conter outros componentes, como potássio ou cálcio, e podem utilizar tampões, como lactato ou acetato, para ficarem semelhantes ao plasma.^(9)^

O solução salina (NaCl 0,9%) é considerado uma solução isotônica, com osmolalidade próxima à do plasma (287mOsm/kg), e contém uma concentração de sódio de 154mEq/L e de cloreto de 154mEq/L, ou seja, a concentração é 1,5 vez maior que a concentração sérica fisiológica de cloreto. Por esse motivo, considera-se o solução salina como solução não balanceada.^(9)^ Como a solução salina normal tem maior concentração de cloreto, as infusões em grande volume podem desencadear acidose hiperclorêmica, também conhecida como acidose hiperclorêmica por diluição, que pode ser explicada pela abordagem de diferença de íons fortes (SID - *strong ion difference*).^(17)^

A SID é definida como a diferença entre cátions e ânions dissociados no plasma.^(17)^ Em um homem saudável de 70kg, a concentração plasmática de sódio e cloreto é de aproximadamente 140mEq/L e 100mEq/L, respectivamente, e o volume de água corporal total é de cerca de 42L. Assim, em indivíduos saudáveis, a concentração de cátions excede a de ânions, resultando em uma diferença de 40mEq/L (SID plasmático =40mEq/L). Nessas condições, as concentrações corporais totais de sódio e cloreto são 5.880mEq e 4.200mEq, respectivamente. Se esse indivíduo receber 10L de solução salina, acrescentará 1.540mEq no sódio plasmático e 1.000mEq no cloreto plasmático, o que resulta em sódio e cloreto corporal total de 7.420mEq e 5.200mEq, respectivamente. A água corporal total também aumentará de 42 para 52L. Assim, o SID é reduzido para 32mEq/L, e a concentração de sódio e cloreto aumenta para 142,7 e 110mEq/L, respectivamente.^(18,19)^ Isso ocorre porque a solução salina contém cátions e ânions fortes na mesma quantidade, por exemplo seu SID é zero. Assim, a infusão de solução salina normal reduzirá o SID do plasma e, desse modo, reduzirá seu pH. Em geral, a acidose é leve a moderada, a variação de excesso de base não é maior que -10mEq/L, e o pH raramente atinge menos de 7,30 após compensação respiratória.^(20)^ Se a infusão salina for interrompida, espera-se que os efeitos sejam transitórios e se revertam em 48 horas.^(21)^

Além da acidose hiperclorêmica, a infusão de grandes quantidades de solução salina pode comprometer a coagulação, a função renal e a resposta imunológica.^(22)^ A coagulopatia dilucional ocorre porque todos os fatores de coagulação serão diluídos pelo volume de solução salina, aumentando o risco de sangramento.^(22)^ Além disso, há cada vez mais evidências demonstrando que a solução salina normal pode afetar a função renal. Em estudos experimentais em animais, demonstrou-se que a hipercloremia induzida pela solução salina diminui o fluxo sanguíneo renal e estimula a vasoconstrição renal.^(23,24)^

As soluções balanceada foram propostas como alternativa para a solução salina.^(25)^ Uma solução pode ser considerada em equilíbrio ideal quando estiver normotônica com SID de 24mEq/L.^(26)^ O que pode ser conseguido removendo-se 24mEq/L de cloreto de uma solução de cloreto de sódio a 0,9% e substituindo essa quantidade por bicarbonato ou ânions orgânicos, que desaparecem rapidamente após a infusão, como o lactato ou o acetato.^(26)^ Considerando os eventos adversos relacionados a uma solução não balanceada descritos anteriormente, as soluções balanceadas podem ser a resposta ideal para a ressuscitação de pacientes críticos.

As soluções balanceadas utilizadas com maior frequência incluem o Ringer simples, Ringer Lactato, Ringer Acetato e Plasma-Lyte. Ringer Lactato foi desenvolvido no início da década de 1930, acrescentando-se lactato de sódio a uma solução de Ringer como tampão, reduzindo sua concentração de cloreto (109mEq/L), quando comparado à solução de Ringer simples (Tabela 1). Ringer Lactato é uma solução levemente hipotônica (273mOsm/kg) e contém potássio e cálcio. A preocupação de que uma infusão de grande quantidade de Ringer Lactato pudesse aumentar o nível plasmático de lactato em pacientes graves levou à substituição do tampão de lactato pelo de acetato, para criar o Ringer Acetato. A composição do Ringer Lactato e Acetato é quase idêntica, com exceção do tampão adicionado (lactato ou acetato). *Plasma-Lyte* é outra solução balanceada com osmolalidade de 295mOsm/L, concentração de sódio de 140mEq/L e concentração de cloreto de 98mEq/L. Outros eletrólitos e tampões que compõem essa solução são potássio, magnésio, acetato e gluconato. Em pacientes com função renal comprometida, deve-se evitar esse tipo de solução, devido ao risco de hipercalemia (Tabela 1).

Foi demonstrado que uma estratégia com restrição de cloreto em pacientes graves estava associada à redução significativa de injúria renal aguda e ao uso de terapia de substituição renal.^(27)^ Além disso, uma grande coorte retrospectiva, com 53.448 pacientes sépticos, demonstrou que a ressuscitação com fluidos balanceados reduz o risco de morte hospitalar em comparação com fluidos não balanceados (risco relativo – RR=0,86; intervalo de confiança de 95% − IC 95%: 0,78-0,94; p=0,001). No entanto, não foram relatadas diferenças significativas na incidência de insuficiência renal aguda, necessidade de terapia de substituição renal, e tempo de internação em unidade de terapia intensiva (UTI) e hospital.^(25)^

No entanto, o nível de evidência para corroborar o uso de soluções balanceadas na prática clínica é baixo.^(28)^ Metanálise recente sugeriu que a ressuscitação com cristaloides balanceados, em comparação com solução não balanceada (solução salina a 0,9%), pode estar associada a menor taxa de mortalidade (*odds ratio* – OR=0,78; IC95%: 0,58-1,05).^(28)^ Nesse contexto, ainda é necessário um grande ensaio clínico randomizado, comparando soluções balanceadas e não balanceadas para ressuscitação, em caso de choque séptico.^(28)^

## COLOIDES

Coloides são definidos como substâncias homogêneas não cristaloides formadas por moléculas grandes ou partículas ultramicroscópicas de uma substância, dispersas através da molécula de uma segunda substância com alto peso molecular.^(29)^ Esses fluidos têm duração relativamente maior e capacidade de expansão intravascular com menores volumes, ou seja, uma pressão oncótica mais alta quando comparada à dos cristaloides. Os coloides não conseguem atravessar a membrana vascular impermeável devido ao seu alto peso molecular.^(29)^

Há dois tipos de coloides: naturais e semissintéticos. A albumina humana em solução salina é a solução coloidal de referência e representa um coloide natural derivado do plasma humano. Coloides semissintéticos, por outro lado, consistem de derivados de três grupos moleculares principais: gelatinas, dextrans e amidos (Tabela 2). Para produzir um coloide, essas moléculas são suspensas em um solvente, que pode ser uma solução salina isotônica ou hipertônica, glicose hipertônica, ou uma solução isotônica balanceada de eletrólitos. A solução salina isotônica é o solvente mais comum utilizado em soluções coloidais. Devido ao escopo dessa revisão, focaremos nas soluções de amido e albumina.

### Hidroxietilamido

O hidroxietilamido (HES), uma solução sintética produzida por meio da manipulação de amilopectina cerosa ou de batata (um polímero multirramificado da glicose), tornou-se um dos expansores plasmáticos coloidais mais utilizados em todo o mundo, principalmente devido a seu custo mais baixo em comparação com a albumina.^(30)^ Atualmente, os HES vêm sendo evitados no tratamento de pacientes graves, especialmente naqueles que apresentam sepse. Dados clínicos recentes indicam que os coloides não melhoram o desfecho dos pacientes e podem ser prejudiciais, dependendo do contexto e do tipo de coloide.^(31,32)^

HES são identificados por três números, por exemplo, 10% HES 200/0,5 ou 6% HES 130/0,4. Sendo classificados de acordo com o peso molecular médio (variação de 70 a 670 kiloDalton) e a frequência de grupos hidroxietil por monômero de glicose (variação de 0,4 a 0,7).^(33)^ O primeiro número indica a concentração da solução, o segundo representa o peso molecular médio em kiloDalton (kDa), e o terceiro e mais significativo é a substituição molar (MS - *molar substitution*).^(33)^

Os HES têm um número variável de resíduos hidroxietil ligados às partículas de glicose anidra no polímero. Essa substituição aumenta a solubilidade do amido em água e, em graus variáveis, inibe a taxa de degradação do polímero de amido pelas amilases.^(33)^ Esses parâmetros são muito relevantes para a farmacocinética dos HES. A meia-vida da solução de amido depende de seu peso molecular, do grau de substituição, e da proporção de grupos hidroxietil no carbono C2, em comparação com o carbono C6 do monômero de glicose.^(34)^ Os grupos hidroxietil na posição do átomo C2 inibem o acesso da alfa-amilase ao substrato de forma mais efetiva do que os grupos hidroxietil na posição C6. Assim, espera-se que os HES produzidos com altas proporções C2/C6 sejam degradados mais lentamente.^(34)^ Em geral, o HES é utilizado para estratégias restritivas de fluidos, devido a uma alta capacidade de expansão plasmática com administração menor de volume.

Um ensaio clínico prospectivo multicêntrico foi realizado para avaliar a frequência de insuficiência renal aguda em casos de sepse grave e choque séptico em pacientes ressuscitados com HES a 6% (200kDa, 0,60; substituição 0,66) ou gelatina fluida modificada 3%.^(35) ^A frequência de insuficiência renal aguda e oligúria, e o pico da concentração de creatinina sérica foram significativamente mais altos no grupo HES em comparação com o grupo gelatina.^(35)^ Nesse estudo, observou-se que a ressuscitação com HES foi um fator de risco independente para insuficiência renal aguda (OR=2,57; IC95%: 1,13-5,83; p=0,026) em pacientes com sepse grave ou choque séptico.^(35) ^Outros estudos avaliaram amidos com peso molecular alto ou intermediário (200 ou 450kDa) e maior grau de substituição molar (0,5 a 0,7), e apresentaram maior incidência de insuficiência renal ou complicações relacionadas a sangramento.^(36)^

Defendeu-se a ideia de que os HES de terceira geração, com menor peso molecular e menor grau de substituição molar, teriam um perfil mais seguro e, portanto, estariam associados a menor incidência de eventos adversos (especialmente complicações por hemorragia e lesão renal aguda). Assim, poderiam ser utilizados para o tratamento de pacientes graves. No entanto, essa hipótese não se confirmou nos ensaios clínicos mais recentes.^(37-39)^ Nesses estudos, a ressuscitação com HES de terceira geração foi associada a maior risco de morte, insuficiência renal aguda e necessidade de terapia de substituição renal, especialmente entre os pacientes sépticos.^(37-39)^ Os resultados relatados foram bastante semelhantes, independente da comparação de amidos derivados da batata ou do milho.^(40)^ Em resumo, a literatura mais recente não corrobora o uso do HES durante a ressuscitação de pacientes com sepse grave ou choque séptico.

### Albumina

As soluções de albumina são utilizadas em todo o mundo para o tratamento de pacientes graves. Uma metanálise de 1998 associou o uso de albumina a alta taxa de mortalidade.^(41)^ Nesse contexto, a segurança do uso da albumina foi questionada até que o estudo SAFE (*Saline versus Albumin Fluid Evaluation*) foi publicado, demonstrando que as soluções de albumina, em comparação com cristaloides, não aumentavam a mortalidade.^(42)^

A administração de albumina pode ser justificada com base em seus efeitos fisiológicos, principalmente ligação e transporte de várias substâncias no sangue (drogas e hormônios, por exemplo); propriedades antioxidantes; modulação de óxido nítrico; e capacidade de tamponamento, que pode ser particularmente relevante para pacientes graves e não apenas para regular a pressão osmótica.^(43)^ Além disso, o nível reduzido de albumina sérica, um achado comum em pacientes graves, está associado a desfechos piores.^(44)^ Por outro lado, há algumas limitações para o uso amplo de soluções de albumina: seu alto custo, risco potencial de transmissão de microrganismos e efeitos alergênicos, quando comparadas com os cristaloides.^(44)^ Até o momento, nenhum ensaio clínico randomizado demonstrou um benefício real para a administração de albumina. Na verdade, pode-se observar que algumas subpopulações, como as vítimas de trauma craniencefálico, podem apresentar maior risco de morte ao receberem soluções de albumina.^(45)^

A análise de um subgrupo de pacientes em choque séptico incluídos no estudo SAFE apresentou uma tendência à redução na mortalidade a favor da albumina, em comparação com a solução salina normal (OR ajustado 0,71; IC95%: 0,52-0,97; p=0,03).^(46)^ Mais recentemente, o estudo ALBIOS (*Volume Replacement With Albumin in Severe Sepsis*) randomizou 1.818 pacientes em sepse grave ou choque séptico para receberem 300mL de albumina a 20% mais cristaloide, ou para receberem apenas cristaloide do momento da randomização até o 28^o^ dia ou a alta da UTI, com meta de manutenção da albumina sérica ≥30g/L.^(47) ^Apesar de mais pacientes terem atingido a meta de PAM em 6 horas no grupo da albumina, quando comparado ao grupo dos cristaloides, nem a taxa mortalidade com 28 dias nem a taxa de mortalidade com 90 dias (RR=0,94; IC95%: 0,85-1,05; p=0,29) diferiram entre os grupos.^(47)^

Podemos concluir que existe a necessidade de um ensaio clínico comparando uma solução de albumina a 4% com cristaloides para ressuscitar pacientes em choque séptico. A solução de albumina, por exemplo, é a única solução coloidal aprovada pelas diretrizes da *Surviving Sepsis Campaign* para pacientes sépticos que não respondem a infusão de cristaloides.^(1)^

## CONCLUSÃO

Não há consenso sobre qual tipo de fluido deve ser utilizado como primeira escolha para ressuscitar pacientes com choque séptico. As evidências mostram que soluções cristaloides, balanceadas ou não, são a escolha mais aconselhável. Soluções de albumina hipo-oncóticas podem ser utilizadas como alternativa para os pacientes que precisam de grande quantidade de fluido durante a fase inicial de ressuscitação. Os hidroxietilamidos não devem ser prescritos para essa população devido a seus efeitos deletérios. São necessárias novas evidências sobre o uso de albumina e soluções balanceadas nessa população de pacientes.
